# Everybody's Page

**Published:** 1889-03-02

**Authors:** 


### Everybody's Page.

Consumption is very frequent among nuns, the life of the cloister, with its frequent fastings and its lack of exercise being favourable to the development of the disease.

An absent-minded doctor, who had recently gone in for some investments in house property, was leaving a prescription with a patient, when he was asked to give directions how the medicine should be taken. "Ah! I forgot," he answered, "One third down, and the balance in two or three years."

"The propaganda of health is the propaganda of morals," says Jules Simon. If a man gets good well-cooked food in a house that is kept clean and airy, if he has a good bed to sleep on, and happy faces around him, he will be less inclined to go to a hot and unhealthy tavern, where he contracts debts, and often picks up blows and disease.

A new application of waste products is the manufacture from cedar-wood pulp of a paper for laying under carpets and wrapping wool, furs, and other things on which moths love to feast. The paper makers get the cedar chips from the pencil manufacturers, and the paper has been voted a great success be everybody but the aforesaid moths, who simply can't bear it.

In Paris they are manufacturing artificial cod-liver oil. It is made from stale and refuse fish, and its connexion with either the cod or its liver is remote and accidental. If these chance to be among the offal, they will be used, but one fish is as good as another to those ingenious Parisians. On the whole, people who like to know what they are swallowing would do well to avoid artificial cod-liver oil.

The reason a glass chimney makes such a difference to the light of a lamp is that it increases the supply of oxygen to the flame by producing a draught. It also concentrates and reflects the heat of the flame. The result of this is that the combustion of carbon is more perfect, and very little is unconsumed. Aimé Argand, the inventor of the Argand lamp, was the first to introduce lamp-glasses. He had been experimenting for some time with various devices for increasing the light of lamps, when he picked up the broken neck of an oil flask that lay on the table near him and, almost without thinking, placed it over the wick. The increased brilliancy of the flame at once struck him, and the lamp-glass was invented.

An unexpected testimony to the value of inoculation comes from South Africa. Mr. G. A. Farini, the explorer of the Kalahari desert, had some oxen bitten by poisonous snakes, and one of his bushmen, it is said, cured them by making incisions round the bite, and rubbing into these a powder made from the dried poison-sacs of other snakes. In a few hours the inflammation caused by the bite disappeared, and the oxen were quite well. A few days afterwards the bushman himself was bitten. He at once inoculated himself in a similar way with the powder, and having extracted from the wound the fangs of the snake that had bitten him, he drank a drop of poison from the virus-sac. He immediately fell into a stupor, that lasted some hours. At first the swelling of the wound increased, but it soon subsided. Next morning he inoculated himself again. By the evening of that day the swelling had disappeared, and two days after he was as well as ever. The favourite antidote is a little lizard of very deadly powers, called N'auboo, which is so valued that a native will give an ox for the dead body of one, to dry and reduce to powder for this purpose; but if he has no N'auboo, he uses the poison of any snake he can get.

"It is," says the *American Druggist*, "rather rough on a doctor's son to be named William, and to have to sneak through the elysium of youth under the opprobrious title of 'the Doctor's Bill.'"

Mexico is thinking of going in for speculation, and doing it thoroughly. A scheme is under consideration for tunnelling the volcano of Popocatepetl, through the wall of the crater, in order to reach the immense sulpbur deposits inside the mountain.

Sir Walter Raleigh was the first European to observe the effect of curare, and in 1595, after the discovery of Guiana, he brought some of it to Europe. It was used by several of the savage tribes of South America to poison their arrows, and some of these mixed with the vegetable poison the venom of serpents to make their weapons more deadly.

Nurses often complain of swollen feet, especially when they first go into hospital. A powder which is much used in the German army for sifting into the shoes and stockings of the foot soldiers might be of service to them. It consists of three parts of salicylic acid, ten parts of starch, and eighty-seven parts of pulverized soapstone. This keeps the feet dry, prevents chafing, and heals any sore spots. The soapstone alone, without the other ingredients, has also been found useful.

Count Romford, who was almost a universal genius, offered, in March, 1796, to fit up a kitchen, constructed on scientific principles, for the Foundling Hospital. So successful was it that the matron of the Foundling said of it after a year's trial, "the saving to the hospital in fuel is very considerable, being about twenty-five chaldrons of coals a year. There were two cooks in the old kitchen, and they had a severe and hot service; one cook in the present kitchen has a very easy one, and the food, particularly the roast beef, is better dressed than formerly."

The photographic camera promises to be of even greater service in medicine than was imagined. It seems to have literally a superhuman faculty of diagnosis. Some time ago a photographer had as a subject a child apparently in good health, with a clear skin and good complexion. When the negative was examined, however, the picture showed the face to be thickly covered with blotches. Within a week the child was covered with the eruption of measles. Another case is recorded when a child's portrait showed spots a fortnight before it was laid up with small-pox, and before any trace of the disease had appeared. It would seem that the sensitive plate of the camera perceived and photographed the eruption before it was visible to the naked eye.

Our own age shows more mercy to drunkenness than was ever displayed before. In classic Greece drunkards were subjected to the severest penalties. In Athens Solon punished them with death; Lycurgus, in Sparta, destroyed the vineyards in his territory, and took every precaution to prevent the transmission of a habit of inebriety from father to child. In Locris wine was permitted only to invalids; and at Mitylene, Pittacus, in opposition to the practice of our modern judges, doubled the punishment of any crime if it had been committed under the influence of wine. In Republican Rome the citizens, both men and women, were forbidden to partake of wine before they had attained the age of thirty. In mediæval times the same severity obtained, and Francis I., though himself no model king, published in 1536 an edict to the effect that everyone found drunk should be imprisoned with a bread and water diet for the first offence, beaten with rods for the second, for the third publicly whipped, and if he then proved incorrigible, punished by having an ear cut off, marked as infamous, and banished.

